# New therapeutic option in genitourinary syndrome of menopause: pilot study using microablative fractional radiofrequency

**DOI:** 10.1590/S1679-45082017AO4051

**Published:** 2017

**Authors:** Márcia Farina Kamilos, Celso Luiz Borrelli

**Affiliations:** 1 Hospital Heliópolis, São Paulo, SP, Brazil.

**Keywords:** Atrophy, Vagina/pathology, Vulva/pathology, Dyspareunia, Radio waves, Lasers, Female urogenital diseases, Atrofia, Vagina/patologia, Vulva/patologia, Dispareunia, Ondas de rádio, Lasers, Doenças urogenitais femininas

## Abstract

**Objective:**

To evaluate the clinical response of patients with symptoms of genitourinary syndrome of menopause after application of microablative fractional radiofrequency in the vagina and vaginal introitus.

**Methods:**

Fourteen patients with symptoms of genitourinary syndrome of menopause underwent three applications of microablative fractional radiofrequency with a 30-day interval, using the Wavetronic 6000HF-FRAXX device and a fractional vaginal electrode. The questionnaires World Health Organization Quality of Life (for quality of life evaluation), Female Sexual Function Index and Quality of Life Adapted Questionnaire in the Domain of Sexual Satisfaction (for sexual function and satisfaction evaluation) were administered before and after the applications (30 to 60 days after the last procedure), in addition to the satisfaction questionnaire after procedure.

**Results:**

There was an increase in almost all dimensions on average in quality of life, with statistical significance only in the health domain. There was a significant improvement in the sexual domains in almost all dimensions. All patients stopped using lubricant during intercourse after treatment. In the satisfaction questionnaire after treatment, we observed that the vast majority felt cured or much better (29% and 64%, respectively, total of 92.6%) and were very satisfied or satisfied (43 and 57%, respectively, total of 100%). The only patient who reported little improvement had an 18-year postmenopausal history and was treatment naïve.

**Conclusion:**

Microablative fractional radiofrequency was effective in treating symptoms of vaginal dryness and dyspareunia, and eliminated the use of vaginal lubricant during the period observed. Since this is a pilot study with a small number of patients, further studies are required to corroborate our findings and evaluate the long-term effects of microablative fractional radiofrequency on the vaginal tissue.

## RESUMO

**Objetivo:**

Avaliar resposta clínica de pacientes com sintomas da síndrome geniturinária da menopausa após aplicação de radiofrequência fracionada microablativa na vagina e no introito vaginal.

**Métodos:**

Quatorze pacientes com sintomas de síndrome geniturinária da menopausa foram submetidas a três aplicações de radiofrequência fracionada microablativa com intervalo de 30 dias, utilizando aparelho Wavetronic 6000HF-FRAXX e eletrodo vaginal fracionado. Foram aplicados os questionários *World Health Organization Quality of Life* (para avaliar qualidade de vida), *Female Sexual Function Index* e *Quality of Life Adapted Questionnaire in the Domain of Sexual Satisfaction* (para verificar função sexual e satisfação) antes e depois das aplicações (30 a 60 dias após último procedimento), além do questionário de satisfação após procedimento.

**Resultados:**

Na qualidade de vida, houve aumento na média em geral, com significância estatística apenas no quesito saúde. No domínio sexual, houve melhora significativa em quase todas as dimensões. Todas as pacientes cessaram o uso de lubrificante na relação sexual após o tratamento. No questionário de satisfação após tratamento, a maioria se sentiu curada ou muito melhor (29 e 64%, respectivamente; total de 92,6%) e estava muito satisfeita ou satisfeita (43 e 57%, respectivamente; total de 100%). A única paciente que relatou pouca melhora tinha história de 18 anos de pós-menopausa e era virgem de tratamento.

**Conclusão:**

Radiofrequência fracionada microablativa foi efetiva em tratar sintomas de ressecamento vaginal e dispareunia, e eliminou o uso de lubrificante vaginal durante o período observado. Por se tratar de estudo piloto com pequena quantidade de pacientes, mais estudos são necessários para corroborar estes achados e avaliar os efeitos a longo prazo da radiofrequência fracionada microablativa no tecido vaginal.

## INTRODUCTION

The term “vulvovaginal atrophy” (VVA) is frequently used to describe symptoms resulting from decreased estrogen and other sexual steroids levels, which are common within the climateric syndrome, comprising vulvar, vaginal, urethral and bladder changes. On the other hand, the term “genitourinary syndrome of menopause” (GSM) has been gaining notoriety since 2012, when the board of the International Society for the Study of Women's Sexual Health (ISSWSH) and the administration board of the North American Menopause Society (NAMS) acknowledged the need to review the terminology for VVA, considering the genitourinary symptoms during the postmenopausal period.^(^ ^1^ ^,^ ^2^ ^)^

Thus, GSM may include, but is not limited to, genital symptoms such as vulvar vestibule and vaginal dryness, burning sensation, discomfort, and vulvovaginal irritation, in addition to sexual symptoms, such as lack of lubrication and dyspareunia, leading to impaired intercourse, and urinary symptoms like urinary urgency, frequent urination (pollakiuria), dysuria and recurrent urinary infections.^(^ ^3^ ^)^

Hypoestrogenism can occur in other situations, such as surgical menopause, or the use of gonadotropinreleasing hormone (GnRH) agonist (*e.g.* in the treatment of endometriosis and leiomyoma); also, in hypothalamic amenorrhea due to excessive exercise, and eating disorders. Moreover it can occur in conditions that affect the production of ovarian estrogen or cause damage to the vaginal epithelium, the vascular supply and the vaginal anatomy such as surgeries, chemotherapy and radiation therapy.^(^ ^1^ ^-^ ^4^ ^)^

Clinically, the genital epithelium becomes thin, pale and dry, and it might cause vaginal restriction and shortening. The mucosa may be less elastic, with a gradual loss of roughness and alteration of the vaginal microbiota, in addition to decreased blood flow. In severe atrophy, the surface of the vestibule and vagina may appear friable, with petechiae and ulcerations, easy bleeding, and even stenosis and tapering of the vaginal fornices. The discomfort associated with VVA can have a significant impact on the overall health and quality of life, but patients not sexually active may go through this period without experiencing most of the symptoms previously mentioned.^(^ ^1^ ^-^ ^7^ ^)^

Hypoestrogenism affects the normal structure and function of genital tissues, largely contributing to the loss of the mucosal elasticity by inducing fusion and hyalinization of collagen fibers, as well as elastin fiber fragmentation. Hydration of the vaginal mucosa is decreased in the dermal layer with reduction of mucopolysaccharides and intercellular hyaluronic acid, which generates a thin stratified epithelium with only basal and parabasal layers.^(^ ^3^ ^,^ ^7^ ^-^ ^10^ ^)^

Recommendation for the initial treatment of symptomatic GSM consists of using long-acting vaginal moisturizers. In moderate to severe VVA, or in those with a milder degree, which did not respond to the use of moisturizers, treatment with replacement of local estrogen at low doses has been considered as the standard treatment. Systemic replacement is not always accompanied by significant improvement of vaginal dryness symptoms, and topical treatment with vaginal estrogen (cream or ring) is best indicated.^(^ ^2^ ^,^ ^3^ ^,^ ^9^ ^,^ ^10^ ^)^

Non-pharmacological approaches are beneficial mainly in women with contraindications to the use of hormones, or those who do not want to use them; but they can also be used as adjuvant or substitutive therapy for all women.^(^ ^7^ ^,^ ^8^ ^)^

Physical methods such as laser and radiofrequency (RF) in the non-ablative, ablative and microablative forms have been used with the purpose of rejuvenating the skin of the face, neck and body. Fractional laser is also used in the vaginal mucosa, aiming to promote neocollagenesis and neoelastogenesis. Studies with clinical, histopathology, electron microscopy as well as immunohistochemistry evaluation have shown satisfactory results on the effects of laser and RF on the regenerative process of the skin. More recently, studies have also shown good results of the effects of laser in the vaginal mucosa.^(^ ^8^ ^,^ ^11^ ^-^ ^17^ ^)^

With RF and laser technologies, a reduction in the recovery period was observed in relation to traditional ablation, despite the need for reapplication in some cases to obtain the same result, however with a lower rate of complications and consistent or more persistent clinical improvement compared to nonablative methods.^(^ ^11^ ^-^ ^24^ ^)^

Radiofrequency is a process of cutting and/or coagulating biological tissues by using a high-frequency alternating current, which instantly raises the intracellular temperature up to 100°C, thus determining cellular membrane expansion and rupture. This phenomenon is known as vaporization, similar to the laser action.

Conventional electrosurgery devices amplify the electrical alternating current provided as 60 cycles/ second (60Hertz) and work in the range of 500,000 (500KHz) to 1,500,000 cycles/second (1.5MHz). By reaching the frequency of 4,000,000 cycles/second (4MHz), the FM radio frequency is obtained - giving rise to the name RF electrosurgery. This technology yields effect into biological tissues like the laser technology - gentle, with no trauma, with precise cut and coagulation, through electromagnetic energy in the megahertz (MHz) frequency.^(^ ^13^ ^)^

Energy fractionation consists of energy distribution at equidistant points, producing microscopic columns of thermal injuries in the epidermis and upper dermis, resulting in microscopic columns of treated tissue and intervening areas of untreated skin, which in turn achieve faster reepithelialization.^(^ ^13^ ^)^

Based on the use of fractional RF on the skin, and of fractionated laser in dermatology and in the genital region, we aimed to study the effects of microablative fractional radiofrequency (MAFRF) with an innovative technique for vaginal application in patients with GSM. Moreover, to evaluate the benefits regarding relief of symptoms as well and the duration of the effects to suggest this technique as a new therapeutic option. A fractional vaginal electrode coupled to the FRAXX platform of the Wavetronic 6000 device was developed for vulvovaginal applications.

## OBJECTIVE

To evaluate the clinical response of patients with symptoms of genitourinary syndrome of menopause after application of microablative fractional radiofrequency in the vagina and vaginal introitus.

## METHODS

A prospective pilot study conducted in the Department of Gynecology at Hospital Heliópolis, from September 2016 to September 2017. A total of 15 patients with GSM complaints were selected and agreed to be treated with microablative fractional radiofrequency (MAFRF) as a therapeutic alternative. One patient withdrew after the first application, for personal reasons.

The inclusion criteria were patients with symptoms of GSM; vaginal, vulvar and/or urinary complaints; perimenopause; surgical menopause; other hypoestrogenic conditions (except for those resulting from chemotherapy, radiation therapy); and cervical citology test negative for cancer within the routine period recommended by the Brazilian Ministry of Health. The exclusion criteria were use of hormone therapy (either systemic or topical) or long-acting moisturizers within the last 60 days prior to the initial assessment; patients with active or recurrent genital infection (*e.g.* genital herpes, candidiasis); patients with human immunodeficiency virus; recurrent urinary tract infection; pelvic radiation therapy or brachytherapy; reconstructive pelvic surgery. Other chronic diseases, such as diabetes, hypertension and deep venous thrombosis were not exclusion criteria.

After history taking and physical examination, patients were selected and instructed about the procedure, and, after giving Informed Consent and authorization for photographic documentation, they answered the following questionnaires: bref version of *World Health Organization Quality of Life* (WHOQoL-BREF), comprising 26 items, which evaluates the general quality of life considering the broad domains physical health, psychological health, social relationships, and environment; *Female Sexual Function Index* (FSFI); and *International Consultation on Incontinence Questionnaire - Vaginal Symptoms* (ICIQ-VS), part of *Quality of Life Adapted Questionnaire in the Domain of Sexual Satisfaction.* Patients were informed that 30 to 60 days after the last MAFRF application, the three questionnaires would be answered again in addition to a questionnaire on post-procedural satisfaction with Likert scale. Physical examination and new assessments could be carried out every 6 months. The clinical outcome was evaluated by analyzing the questionnaires.

The study was approved by the Research Ethics Committee under number 1769977, CAAE 58353416. 2.0000.5449 and conducted according to the guidelines recommended by the 2000 Declaration of Helsinki, updated in 2008.

### Application technique

**Figure 1:**
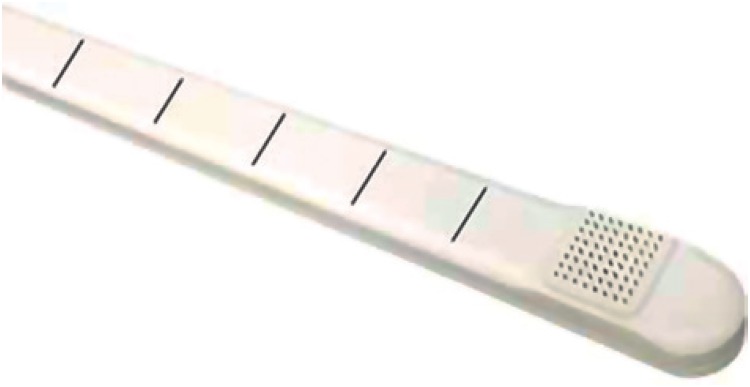
Vaginal pen with 64 microneedles

No anesthetic agent was required for the vaginal procedure. In the vestibule and vaginal opening, 10% lidocaine spray was applied 3 minutes prior to the procedure. The Wavetronic 6000 Touch device is used with the Megapulse HF FRAXX system (Loktal Medical Electronics, São Paulo, Brazil), equipped with an electronic circuit of energy fractionation, connected to a vaginal pen with 64 microneedles 200μ in diameter and 1mm in length, mounted on a Teflon body and divided into an eight-column matrix with eight needles each (Figure 1). When pressing the activator pedal, the 64 needles are not energized at the same time, and energy delivery is randomized into columns of eight needles in a pre-set sequence in such a way that two underlying columns do not shoot in sequence, thus preventing thermal summation of columns (exclusive randomic fractionated shot control “Smart Shoot”).

**Figure 2:**
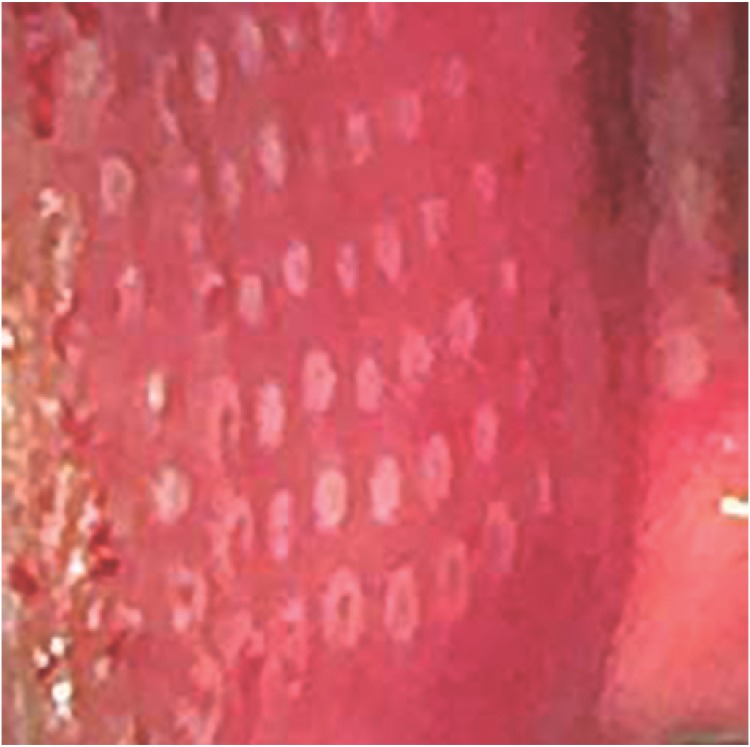
Microablations in the vestibule mucosa

**Figure 3:**
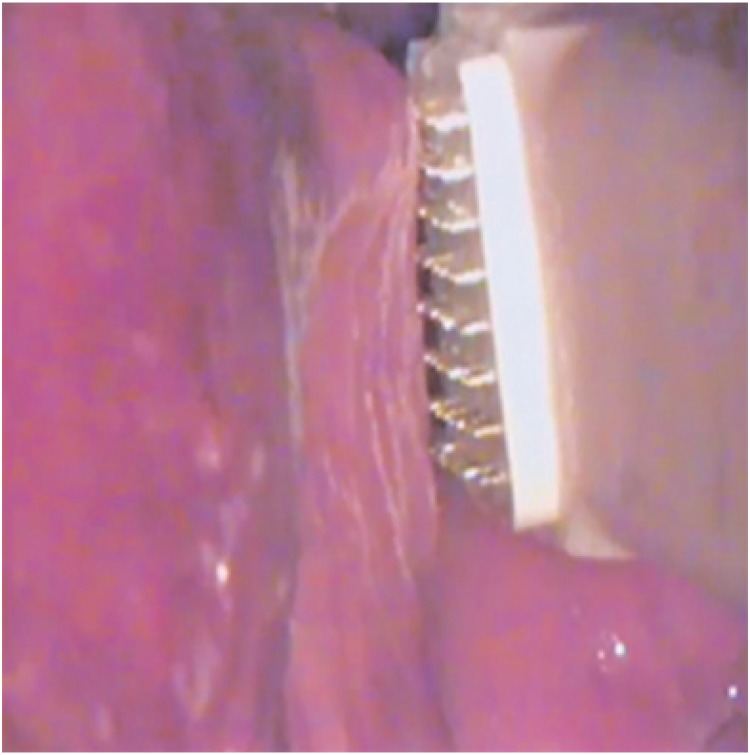
The electrode parallel and lightly touching the mucosa

This allows cooling between the points and preservation of the tissues adjacent to the vaporized points for neocolagenesis and neoelastogenesis to take place through fibroblast stimulation. Each shot of the pen performs 64 microablations in the mucosa (Figures 2 and 3).

### Vaginal/Introitus Application

Three MAFRF applications were performed in the vagina/vaginal introitus, with a 28 to 40-day interval between each application. The following technique was performed: patient in the lithotomy position; placement of the disposable vaginal speculum, antisepsis with 0.2% aqueous chlorhexidine, and cleaning with sterile 0.9% saline solution, removing excess vaginal contents with gauze. A sequential emission of MAFRF was applied on all vaginal walls under direct vision and moving the vaginal speculum as necessary. In the vestibule, the application was limited to the vaginal introitus, not including the clitoris, clitoral foreskin and labia minora. The electrode was always kept parallel and lightly touching the mucosa at each shot. The average procedure time was 15 to 20 minutes.

For the post-treatment care, the use of 5% dexpanthenol solution in the vaginal opening was recommended two to three times a day, for 2 to 5 days, and no intercourse for 10 days.

### Statistical analysis

Data are presented as mean and standard deviation, median or percentages. The significance level was set at 0.05, corresponding to a 95% confidence interval. The Student's *t* test was used for dependent samples, and significant differences were analyzed (p<0.05).

## RESULTS

Fourteen women were followed up and the questionnaires were administered comparing the two periods - pre-and post-treatment. The main complaints of patients were vaginal dryness (100%); need for lubricant during intercourse (86%); dyspareunia (50%); urinary urgency (29%); mild urinary incontinence (29%); nocturia (29%); urinary tract infection after sexual intercourse (7%); and bleeding during intercourse (7%).

Considering the WOHQoL, we found an increase in almost all dimensions on average, with statistical significance only in the health domain (p=0.0401) (Table 1).

**Table 1:** Dimensions of World Health Organization Quality of Life Questionnaire* of women with symptoms of genitourinary syndrome of menopause

Dimensions	Before	After	p value
General	67.9 (20.6)	76.8 (11.9)	0.1365
Health	64.3 (23.4)	71.4 (19.3)	0.0401
Physical	64.0 (11.0)	62.5 (10.2)	0.4807
Psychological	70.2 (20.9)	78.9 (11.4)	0.0889
Social relations	66.7 (21.9)	78.0(6.2)	0.0832
Environment	64.1 (20.9)	67.9 (13.2)	0.3852

Mean and standard deviation, Student's *t* test; significant difference p<0.05.

* Development of the World Health Organization WHOQOL-BREF quality of life assessment. The WHOQOL Group. Psychol Med. 1998;28(3):551-8.

In the FSFI, we noticed significant improvement in the overall total (p=0.0065), in almost all dimensions (desire, arousal, lubrification, satisfaction and pain), except for excitation and orgasm (Table 2).

**Table 2:** Dimensions of Female Sexual Function Index Questionnaire* of women with symptoms of genitourinary syndrome of menopause

Dimension	Before	After	p-value
Desire	3.0 (1.0)	4.1 (0.6)	0.0019
Arousal	3.6 (1.4)	4.1 (1.4)	0.3934
Lubrication	2.9 (1.4)	4.7 (1.6)	0.0010
Orgasm	4.0 (1.5)	4.7 (1.7)	0.1106
Satisfaction	3.9 (1.7)	5.3 (0.9)	0.0032
Pain	2.9(1.7)	4.9 (1.8)	0.0071

Mean and standard deviation, Student's *t* test, significant difference p value <0.05.

* Rosen R, Brown C, Heiman J, Leiblum S, Meston C, Shabsigh R, et al. The Female Sexual Function Index (FSFI): a multidimensional self-report instrument for the assessment of female sexual function. J Sex Marital Ther. 2000; 26(2):191-208.

In the ICIQ-VS, there was a significant improvement in five questions, with statistical significance in the overall total (p=0.0001). All patients stopped using lubricant during intercourse after treatment (Table 3).

**Table 3:** Dimensions of *International Consultation on Incontinence Questionnaire - Vaginal Symptoms* * of women with symptoms of genitourinary syndrome of menopause

Dimension	Before	After	p value
Q1 Difficulty in vaginal intercourse	2.6 (1.2)	1.1 (1.3)	0.0024
Q2 Discomfort or pain in vaginal penetration	2.8 (1.5)	0.9 (1.3)	0.0020
Q3 Itching, burning, irritation in vagina or around	1.2 (1.5)	0.3 (0.6)	0.0703
Q4 Is your vagina very dry?	3.4 (0.9)	0.7 (0.8)	0.0001
Q5 Satisfaction with the appearance of your vulva	1.1 (1.2)	0.5 (1.0)	0.3203
Q6 Use of lubricant	2.6 (1.8)	0.4 (0.9)	0.0039
Q7 How vaginal symptoms interfere with your life?	2.8 (0.9)	0.5 (0.8)	0.0001
Q8 Satisfaction with sexual life in general	2.1 (1.7)	1.0 (0.8)	0.0845

Mean and standard deviation, Student's t test; significant difference p value <0.05.

* Adapted [with eight questions] from: Tamanini JT, Almeida FG, Girotti ME, Riccetto CL, Palma PC, Rios LA. The Portuguese validation of the International Consultation on Incontinence Questionnaire-Vaginal Symptoms (ICIQ-VS) for Brazilian women with pelvic organ prolapse. Int Urogynecol J Pelvic Floor Dysfunct. 2008;19(10):1385-91.

**Figure 4:**
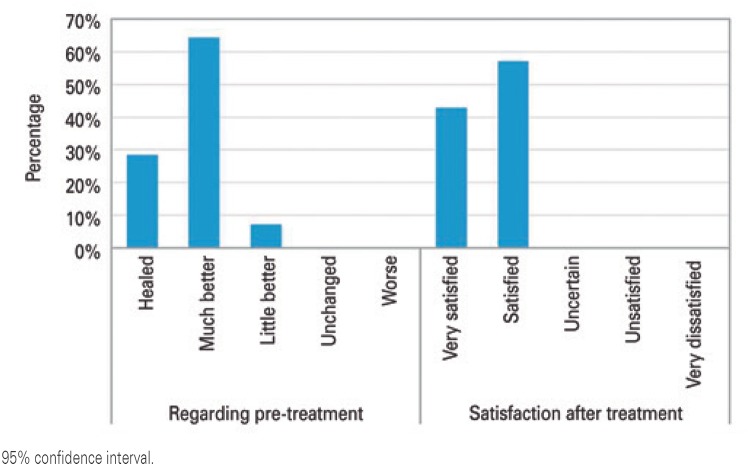
Responses to the satisfaction survey

In the Satisfaction questionnaire after treatment, we observed that the majority felt cured or much better (29% and 64%, respectively; total of 92.6%) and were very satisfied or satisfied (43% and 57%, respectively; total of 100%). The only patient who reported little improvement had an 18-year postmenopausal history and was treatment naïve (Figure 4).

## DISCUSSION

Approximately 40 to 50% of women in physiological menopause may have signs and symptoms of GSM. Early diagnosis and active intervention can prevent the appearance of moderate to severe atrophies, as well as sequelae. Alternative therapies, whether or not associated with local hormone therapy, may contribute to a more complete and appropriate approach to each patient's situation.

In a study on periorbital rejuvenation, the same Wavetronic HF FRAXX device, version 5000, was successfully used, calibrated at 46W power and 60ms current time. This is the active current time during which the skin is exposed to heat, corresponding to 338mJ/point, so that the thermal lesion is similar to that of a fractionated CO^2^ laser, considering the sufficient amount of energy (345mJ) for a safe treatment.^(^ ^13^ ^)^

Since this study was conducted on the skin, which is keratinized and offers more resistance to electromagnetic wave penetration in relation to the mucosa (not keratinized), we decided to use less energy. The device was set at 45W power and a low energy treatment level, 40ms, which is the current time in milliseconds of each eight-needle column, corresponding to 225mJ per point.

In a pilot study in 2014, Salvatore et al. assessed 50 patients with GSM dissatisfied with local estrogen therapy, who received three vaginal applications of CO_2_ laser for 12 weeks. Symptoms were assessed before and after the procedure, using quality of life and sexual function questionnaires. The authors pointed out effectiveness of the treatment proposed as regards the significant improvement of symptoms of GSM among postmenopausal women, and suggested further studies.^(^ ^16^ ^)^

In the present pilot study, whose design is similar to that of Salvatore et al.^(^ ^16^ ^)^ we used MAFRF in 15 patients with GSM and found analogous results. For our group of patients, this therapy was very effective especially in the treatment of vaginal dryness and dyspareunia, eliminating the use of lubricants during the follow-up period.

In another important *ex-vivo* study, Salvatore et al. compared the effects of microablative fractionated CO_2_ laser in the vaginal mucosa of postmenopausal patients using histologic analyses by means of either electronic or light microscopy (hematoxylin and eosin staining), before and after treatment. The authors concluded it could be demonstrated for the first time that fractionated CO_2_ laser was capable of producing vaginal connective tissue remodeling with vaginal mucosa reconstitution.^(^ ^8^ ^)^

The limitations of the present study are related to the small sample size and to the fact that it is based on subjective assessments. This has motivated us to start another research project to assess the presumed histological remodelling of the vaginal mucosa by means of biopsies before and after treatment with three MAFRF applications.

As advantages of the use of MAFRF in the vaginal mucosa in comparison to fractioned CO_2_ laser, we point out that the application is performed under direct vision and using a vaginal speculum, thus facilitating the treatment on the vaginal walls and preventing shot overlapping; in addition, the method is easy to learn and less costly.

The procedure proved to be well tolerated, with occasional reports of mild discomfort; patients recovered fast and microablation was no longer visible usually within 3 to 5 days after the application. The adverse effects observed were not significant and none of the participants had any long-term or permanent side effects after the procedure. Most patients reported improvement of the symptoms of dryness and dyspareunia from the first application.

## CONCLUSION

Microablative fractional radiofrequency was effective in treating symptoms of vaginal dryness and dyspareunia, and eliminated the use of vaginal lubricant during the period observed. Since this is a pilot study with a small number of patients, further studies are required to corroborate our findings and evaluate the long-term effects of microablative fractional radiofrequency on the vaginal tissue.
